# Effects of a Nurse‐Led Multicomponent Exercise Intervention for Haemodialysis Patients With Possible or Confirmed Sarcopenia: A Cluster Randomised Trial

**DOI:** 10.1002/nop2.70651

**Published:** 2026-10-30

**Authors:** Genlian Cai, Liuqian Zhang, Yujiao Zhang, Jing Zhang, Jinjin Zhou, Bingxin Zhan, Qi Guo, Jinping Ying

**Affiliations:** ^1^ Department of Nursing, The First Affiliated Hospital Zhejiang University School of Medicine Hangzhou Zhejiang Province China; ^2^ Kidney Disease Center, The First Affiliated Hospital Zhejiang University School of Medicine Hangzhou Zhejiang Province China

**Keywords:** chronic kidney failure, exercise, nurse‐led intervention, renal dialysis, sarcopenia

## Abstract

**Aims:**

To assess the potential effects, safety and feasibility of a nurse‐led multicomponent exercise intervention on skeletal muscle function and quality of life in haemodialysis patients with possible or confirmed sarcopenia.

**Design:**

A cluster randomised trial with clusters defined by fixed haemodialysis schedules.

**Methods:**

A total of 56 eligible participants were enrolled and allocated through cluster randomisation. The intervention group (*n* = 28) received a 12‐week nurse‐led multicomponent exercise intervention in addition to standard care, whereas the control group (*n* = 28) received standard care. The primary outcomes included skeletal muscle mass index (SMI), handgrip strength, gait speed and short physical performance battery (SPPB) scores. Secondary outcomes included scores on the Kidney Disease Quality of Life 36‐Item Short‐Form Survey (KDQOL‐36) and C‐reactive protein levels. Handgrip strength, gait speed, SPPB scores and KDQOL‐36 scores were assessed at baseline and at Weeks 4, 8 and 12 during the intervention period, whereas SMI and C‐reactive protein were assessed at baseline and Week 12. An intention‐to‐treat analysis was employed, and the effects of the intervention were evaluated using linear mixed models.

**Results:**

Of the 56 participants, 50 completed the study. The intervention group showed favourable trends in handgrip strength, gait speed and SPPB scores, with moderate‐to‐large effect sizes; however, these differences were not statistically significant after Bonferroni correction. Furthermore, no significant between‐group differences were found in skeletal muscle mass index, health‐related quality of life, or C‐reactive protein levels. No serious intervention‐related adverse events were reported.

**Conclusions:**

The nurse‐led multicomponent exercise intervention was safe and feasible and showed favourable trends in muscle strength and physical function, although statistically significant between‐group differences were not retained after correction for multiple comparisons.

**Implications for Patient Care:**

A nurse‐led, multicomponent exercise intervention may have the potential to increase muscle strength and physical function in haemodialysis patients with possible or confirmed sarcopenia.

**Impact:**

What problem did the study address? This study assessed the potential effects, safety, and feasibility of a nurse‐led, multicomponent exercise intervention designed for haemodialysis patients with possible or confirmed sarcopenia.

What were the main findings? A nurse‐led exercise intervention appeared safe and feasible and may help to improve muscle strength and physical function in haemodialysis patients with possible or confirmed sarcopenia. However, future studies with larger sample sizes and longer follow‐up periods are needed to assess the long‐term effects.

Where and on whom will the research have an impact? The results of this study may inform the development and future implementation of nurse‐led exercise interventions for haemodialysis patients with sarcopenia.

**Patient and Public Contributions:**

Patients and family members were invited to collaborate with healthcare professionals in designing a nurse‐led, multicomponent exercise intervention programme. Their contributions helped improve the feasibility and practicality of the exercise intervention.

**Reporting Method:**

The CONSORT 2025 checklist was used in the reporting of the study.

**Trial Registration:**

This trial was registered at www.chictr.org.cn (registration number: ChiCTR2200065863)

## 1 Introduction

Chronic kidney disease (CKD) is a globally recognised public health issue. Once the disease progresses to end‐stage renal disease (ESRD), patients require conservative treatment or renal replacement therapy (including dialysis or kidney transplantation) to survive. By 2023, an estimated 4.59 million people worldwide were estimated to be receiving renal replacement therapy (GBD 2023 Kidney Failure with Replacement Therapy Collaborators 2025). Haemodialysis (HD) remains the primary replacement therapy for patients with ESRD, accounting for approximately 90% of all dialysis recipients (Bello et al. 2022). However, HD only partially replaces kidney function, and patients undergoing prolonged HD are susceptible to complications such as malnutrition, sarcopenia and frailty (Bello et al. 2022).

Sarcopenia is a progressive, systemic syndrome characterised by the loss of skeletal muscle mass, strength and function (Sayer et al. 2024). Factors such as chronic inflammation, oxidative stress, metabolic acidosis, protein‐energy wasting and reduced physical activity contribute to a high prevalence of sarcopenia among HD patients (Wang et al. 2022). Two meta‐analyses have reported prevalence estimates of 26%–29% in this population (Duarte et al. 2024; Shu et al. 2022). Moreover, sarcopenia significantly increases the risk of adverse health outcomes including fractures, frailty, reduced quality of life and even mortality, in HD patients (Ribeiro et al. 2022; Shu et al. 2022; Wang et al. 2022). Therefore, it is essential to implement effective management and intervention strategies to prevent or mitigate the adverse outcomes experienced by HD patients with sarcopenia.

## 2 Background

Currently, there is no specific pharmacological treatment for sarcopenia in clinical practice. Exercise training is widely regarded as a safe, cost‐effective and sustainable first‐line intervention for preventing and managing sarcopenia (Dent et al. 2018; Sayer et al. 2024). Accumulating evidence has supported the benefits of exercise interventions in HD patients with sarcopenia. A previous study reported that combined aerobic and resistance exercise significantly improved the 6‐min walking distance in this population (Zhang et al. 2025). A meta‐analysis showed that resistance exercise improved handgrip strength in HD patients with sarcopenia, whereas combined aerobic and resistance exercise had favourable effects on skeletal muscle mass index (SMI) (Li, Li, et al. 2024). Another study further confirmed the beneficial effects of resistance exercise on muscle function and strength in HD patients (Li, Ma, et al. 2024).

Multicomponent exercise generally combines three or more exercise modalities, such as resistance, aerobic, balance and flexibility exercise. Compared with single‐modality exercise, multicomponent exercise may provide broader benefits by targeting multiple domains of physical function, including muscle strength, mobility, balance and flexibility, thereby contributing to improved quality of life in patients with sarcopenia (Bernabei et al. 2022; Izquierdo et al. 2025; Sayer et al. 2024). However, most previous studies in HD patients with sarcopenia have focused on resistance exercise alone or combined aerobic and resistance exercise, whereas evidence regarding multicomponent exercise in this population remains limited.

Nurses constitute the largest group of healthcare professionals and are well positioned to deliver continuous, patient‐centred care. A nurse‐led, evidence‐based model of care has emerged as a promising approach for improving quality of life, symptom management and clinical outcomes in patients with chronic conditions (Clarke et al. 2024). A recent meta‐analysis showed that nurse‐led interventions improved care quality, medication adherence, self‐management capabilities and self‐efficacy, and reduced the burden of multimorbidity in adults and older adults. These interventions were also associated with improvements in physiological indicators, including blood pressure and glycaemic control (Ruksakulpiwat et al. 2025). In patients with CKD, nurse‐led care interventions have been shown to improve quality of life, sleep patterns, pain and fatigue (Arooj et al. 2025). Furthermore, a prospective observational cohort study reported that a nurse‐led home‐based exercise programme significantly improved short physical performance battery (SPPB) scores, 10‐repetition sit‐to‐stand‐to‐sit 10‐s test (STS10) scores, and 6‐min walking test scores in patients with CKD (Junqué‐Jiménez et al. 2023). Nevertheless, evidence regarding nurse‐led exercise interventions for HD patients with sarcopenia remains limited.

### 2.1 Aim and Objectives

This study aimed to assess the potential effects, safety and feasibility of a 12‐week nurse‐led, multicomponent exercise intervention for HD patients with possible or confirmed sarcopenia. The primary objectives were to determine the effects of the intervention on handgrip strength, gait speed, SPPB scores and SMI. The secondary objectives were to assess its impact on KDQOL‐36 scores and C‐reactive protein levels.

## 3 Methods

### 3.1 Study Design

This was a 12‐week cluster randomised controlled trial. The present report followed the Consolidated Standards of Reporting Trials (CONSORT) guidelines.

### 3.2 Participants

Participants were recruited from a single HD unit between September and October 2024, with follow‐up assessments continuing until February 2025. Trained HD nurses approached eligible patients, and those who provided informed consent were enrolled in the study until the target sample size was reached. The inclusion criteria were as follows: aged 18–80 years; received HD three times a week for ≥ 3 months; and had a diagnosis of possible or confirmed sarcopenia according to the 2019 criteria of the Asian Working Group for Sarcopenia (AWGS) (Chen et al. 2020). The exclusion criteria were as follows: inability to undergo bioimpedance testing (e.g., amputees or pacemaker recipients); inability to walk independently; cognitive impairments; severe visual or hearing impairments; exercise contraindications; and regular exercise habits or participation in any rehabilitation training within the past 3 months.

The following sarcopenia diagnostic criteria (Chen et al. 2020) were utilised: ① low muscle strength (handgrip strength < 28 kg for males, < 18 kg for females); ② low physical function (SPPB scores ≤ 9 or 6‐m gait speed < 1.0 m/s; or 5‐repetition sit‐to‐stand test duration ≥ 12 s) and ③ low skeletal muscle mass (as measured via bioelectrical impedance analysis: SMI < 7.0 kg/m^2^ for males and < 5.7 kg/m^2^ for females). If either the low muscle strength or low physical function criterion was met, the participant's sarcopenia was considered possible; if either the low muscle strength or low physical function, along with the low skeletal muscle mass criteria, was met, the participant's sarcopenia was considered confirmed.

### 3.3 Sample Size

Handgrip strength served as the primary indicator for the diagnosis of sarcopenia (Chen et al. 2020). A previous network meta‐analysis reported that combined aerobic and resistance training significantly improved handgrip strength in HD patients with sarcopenia, with a large effect size (SMD = 3.50; 95% CI: 3.13–3.87) (Li, Li, et al. 2024). Therefore, for this study, the sample size was calculated using Cohen's *d* = 0.80 to represent a large effect size, with a significance level of 0.05 and a power of 0.80. Using statistical software G*Power (version 3.1.9.4), the estimated sample size was 52 participants (26 per group). However, considering an anticipated 10% dropout rate, 56 participants were recruited for the study.

### 3.4 Randomisation and Blinding

Participants were allocated by cluster randomisation based on fixed weekly HD schedules to avoid contamination between groups. No individual stratification by dialysis day was performed. Instead, according to their fixed weekly HD schedules, participants were divided into two clusters: Cluster A (Monday, Wednesday and Friday) and Cluster B (Tuesday, Thursday and Saturday).

Prior to enrolment, an independent research coordinator who was not involved in participant recruitment, outcome assessment, or intervention implementation performed a simple random coin toss to assign Cluster A to either the intervention or control group, with Cluster B automatically assigned to the opposite group. The results of the group allocation were sealed in opaque envelopes and were not disclosed until all baseline assessments had been completed.

As this was a cluster design without individual‐level stratification, unmeasured confounding related to HD scheduling could not be fully excluded. To minimise data bias, data collectors did not participate in the implementation of the exercise intervention programme.

### 3.5 Study Interventions: Multicomponent Exercise Intervention

#### 3.5.1 Development of the Multicomponent Exercise Intervention

Through a review of the literature (Battaglia et al. 2024; Dent et al. 2018; Li, Li, et al. 2024; Sayer et al. 2024) and with reference to the Global Renal Exercise Network (https://grexercise.kch.illinois.edu/) and clinical practice guidelines for exercise and lifestyle in patients with CKD (Baker et al. 2022), evidence was extracted to draft a preliminary multicomponent exercise intervention. A meeting of experts was convened, involving rehabilitation physicians, nephrologists, nursing management specialists and HD nurses, to evaluate the programme draft. On the basis of feedback from the experts, we revised the preliminary intervention programme and finalised the draft. The programme includes aerobic exercise on non‐HD days, as well as flexibility exercises and upper and lower limb resistance exercises on HD days. It is straightforward to learn and requires no specialised equipment. To facilitate participant practice, the research team produced instructional videos of the multicomponent exercise intervention and created exercise log manuals.

#### 3.5.2 Implementation of the Multicomponent Exercise Intervention

The intervention group received a 12‐week nurse‐led multicomponent exercise intervention in addition to standard care. The intervention team consisted of four HD nurses with 10 years of experience, one nephrologist and one rehabilitation physician. The nephrologist was responsible for comprehensive participant assessment and identification and management of adverse events. The rehabilitation physician provided guidance and training on exercise techniques and movement standards prior to the initiation of the intervention. After receiving training and passing the assessment, four HD nurses began implementing the intervention programme; comprehensive details are presented in Table 1. To enhance adherence to the programme, participants were provided with small daily items, including towels and soap, as incentives at weeks 4 and 8 intervals.

**TABLE 1:** Nurse‐led multicomponent exercise intervention programme.

Time point	Exercise component	Detailed implementation	Duration	Frequency	Intensity
Before every exercise	Pre‐exercise assessment	Blood pressure, pulse rate, and self‐reported physical condition were monitored	—	—	—
Non‐HD days	Aerobic exercise	Warm‐up → main exercise → cool‐down. During the main exercise phase, participants were instructed to walk at a comfortably fast pace while actively swinging their arms. The duration started at 5–10 min/session in Week 1 according to tolerance; increase by approximately 5 min every 1–2 weeks if Borg RPE remains 11–13 and no adverse symptoms occur; target ≥ 20 min/session, progressing to ≥ 30 min/session when tolerated	Initial 5–10 min → ≥ 30 min	3 times/week	Borg 11–13
HD days	Flexibility exercises	Prior to engaging in upper limb resistance exercise. Neck/arm stretches, shoulder rotation, chest/lateral stretches, leg stretches; new movements were added when participants could comfortably hold each position for 10–20 s and complete at least 3 repetitions	10 min	3 times/week	Borg 11–13
HD days	Upper‐limb resistance exercises	After flexibility exercises, before HD treatment, participants performed three Thera‐Band upper‐limb exercises: upper‐limb abduction, elbow flexion and side‐arm raise. Each movement was repeated 10–15 times per set, with a 10‐s hold at the target position	15–20 min	3 times/week	Borg 11–13
HD days	Lower‐limb resistance exercises	Within the first 2 h of HD, participants performed lower‐limb resistance exercises after a 5‐min warm‐up. The programme included ankle dorsiflexion, straight‐leg raise, hip abduction A, and hip abduction B. Each movement was repeated 10–15 times per set, with a 10‐s hold at the target position, followed by approximately 5 min of cool‐down activities	15–20 min	3 times/week	Borg 11–13

*Note:* 1. Exercise withholding criteria: Exercise was withheld if SBP ≥ 180 mmHg, DBP ≥ 110 mmHg, pulse ≥ 110 or ≤ 50 bpm, missed dialysis, or severe discomfort occurred. 2. Aerobic exercise: complete ≥ 20 min; patient/family members logged duration and Borg; nurses reviewed exercise logs on dialysis days and adjusted the progression according to symptoms, adherence and safety assessments. 3. Flexibility exercises complete ≥ 10 min. 4. Resistance exercises were performed using Thera‐Band resistance bands, participants advanced to a band with a higher resistance level once they could comfortably perform three to five sets, increasing the exercise load; to count as completed, resistance exercises for the upper and lower limbs must be performed for at least 15 min each. 5. Flexibility and resistance exercises were demonstrated and supervised by trained haemodialysis nurses, who recorded exercise duration, sets, Borg score and adverse events.

Abbreviations: DBP, diastolic blood pressure; HD, haemodialysis; RPE, rating of perceived exertion; SBP, systolic blood pressure.

#### 3.5.3 Standard Care

All participants in the control group received standard care, including routine HD treatment, medication and routine checkups. In addition, they were provided with dietary guidance to ensure adequate protein and energy intake and received education on the prognosis of sarcopenia and current intervention methods. At the end of the study, the researchers provided participants in the control group with videos demonstrating multicomponent exercises, resistance bands, exercise log manuals, and guidance on performing multicomponent exercises.

### 3.6 Outcome Measures

The primary outcomes in this study included handgrip strength, SPPB scores, gait speed and SMI. Secondary outcomes included scores on the Kidney Disease Quality of Life 36‐Item Short‐Form Survey (KDQOL‐36) and C‐reactive protein levels. Handgrip strength, SPPB scores, gait speed and KDQOL‐36 scores were assessed at baseline and at Weeks 4, 8 and 12 during the intervention period. SMI and C‐reactive protein levels were measured at baseline and Week 12. Sociodemographic and disease‐related data were collected only at baseline.

#### 3.6.1 Primary Outcomes

##### 3.6.1.1 Handgrip Strength

Handgrip strength was assessed using a CAM‐RYEH101 electronic handgrip dynamometer (produced domestically by Guangdong Xiangshan Weighing Apparatus Group Co. Ltd.) before HD. During testing, the participants were seated with their shoulders adducted, their elbows flexed at 90°, and their forearms in a neutral position. Three measurements were taken, and the mean value was recorded (Sousa‐Santos and Amaral 2017). Participants with autologous arteriovenous fistulas performed the test with the limb without the fistula, whereas those with central venous catheters used their dominant hand (Cai et al. 2022).

##### 3.6.1.2 SPPB Scores and Gait Speed

The Short Physical Performance Battery (SPPB), developed by Guralnik et al. (1994), is a comprehensive assessment tool for physical function. It comprises three components: a standing balance assessment (including side‐by‐side, semi‐tandem and tandem stances), a four‐metre gait speed test and a five‐repetition sit‐to‐stand test. Each component is scored from 0 to 4 points, yielding a total score ranging from 0 to 12; higher scores indicate better physical performance. The gait speed test measures how long it takes participants to walk 4 m from a standing start and at a normal pace, without accelerating or decelerating. The test was performed at least twice, and the average time was recorded (Chen et al. 2020).

##### 3.6.1.3 SMI

SMI was measured 15 min after the completion of HD using the InBody S10 Biospace Multi‐Frequency Bioelectrical Impedance Body Composition Analyser (Wang et al. 2023).

#### 3.6.2 Secondary Outcomes

##### 3.6.2.1 KDQOL‐36 Scores

Health‐related quality of life was assessed using the Chinese version of the KDQOL‐36^TM^. This instrument is a reliable and valid tool for evaluating the quality of life of patients with kidney disease. It comprises a general health measure and three subscales specific to kidney disease (covering the burden of kidney disease, kidney disease symptoms and problems, and the effects of kidney disease on daily life). The Chinese version of the KDQOL‐36^TM^ has demonstrated good reliability, with Cronbach's alpha coefficients ranging from 0.69 to 0.78 (Tao et al. 2014).

##### 3.6.2.2 C‐Reactive Protein

C‐reactive protein is a widely recognised biomarker of inflammation. Patients who undergo HD frequently present with persistent microinflammatory conditions, which are implicated in the deterioration of skeletal muscle strength, mass and function via multiple mechanistic pathways (Meléndez Oliva et al. 2022; Wang et al. 2022). Blood samples were obtained at baseline and Week 12, before the first HD session of the corresponding week, and were analysed using a particle‐enhanced immunoturbidimetric assay.

#### 3.6.3 Exercise Adherence

Exercise adherence was defined as the proportion of completed exercise sessions compared to the total number of planned sessions. The total number of sessions was calculated as follows: 12 weeks × 3 sessions per week × 4 exercise components (aerobic, upper limb resistance exercise, lower limb resistance exercise and flexibility exercise), resulting in 144 sessions per participant over the 12‐week period. For those who left midway, the proportion of the total planned number of exercise sessions they completed was calculated.

#### 3.6.4 Adverse Events

The expected adverse events included cardiovascular complications and musculoskeletal injuries, such as hypotension, hypertension, arrhythmia, chest tightness, dyspnoea, muscle soreness, cramps, joint pain and headache. All adverse events were assessed by team physicians.

### 3.7 Statistical Analysis

The data were analysed with R version 4.4.2. Baseline data for both groups were subjected to descriptive analyses. Continuous variables with a normal distribution are expressed as the mean (standard deviation), whereas non‐normally distributed continuous variables are presented as the median and interquartile range (IQR). Comparisons between groups were performed with the independent *t*‐test and the Mann–Whitney *U* test. Categorical variables are reported as frequencies and percentages, with group comparisons performed with the chi‐square test or Fisher's exact test.

Intervention effects were evaluated with a linear mixed‐effects model (LMM) in accordance with the intention‐to‐treat (ITT) principle. The outcome variables in the models included the primary and secondary outcome measures. Fixed effects included group, time, and their interaction term. Random effects comprised participant identification numbers. Baseline age, sex and dialysis duration were incorporated as covariates for adjustment. Two candidate models were considered: a random‐intercept model and a random‐intercept plus random‐slope model. Because the random‐slope models failed to converge for the outcomes, the final analyses used random‐intercept models. Model fit was summarised using AIC, BIC, marginal *R* ^2^, conditional *R* ^2^, ICC and residual diagnostics.

Missing outcome data were handled using likelihood‐based estimation in the LMMs without imputation, under the assumption that data were missing at random. Missing data patterns were similar between the two groups and did not differ significantly.

Bonferroni correction was applied to the ten primary comparisons (handgrip strength, gait speed and SPPB scores at three time points each, plus SMI at one time point). All tests were two‐sided. For the primary outcome comparisons, statistical significance was determined using Bonferroni‐adjusted *p* values, with adjusted *p* < 0.05 considered statistically significant. Raw *p* values and 95% confidence intervals were reported to describe estimates before multiplicity correction. Secondary outcomes were exploratory, were analysed without multiplicity correction, and were interpreted descriptively based on raw *p* values and 95% confidence intervals. Cohen's *d* was used to evaluate effect sizes, with values of 0.2, 0.5 and 0.8 representing small, moderate and large effects, respectively (Panjeh et al. 2023). Finally, per‐protocol (PP) analyses were conducted as sensitivity analyses to evaluate the robustness of the primary and secondary outcome findings.

### 3.8 Ethical Considerations

This study was approved by the Institutional Review Board of the First Affiliated Hospital of Zhejiang University School of Medicine (approval nos. 2022‐437; 2024‐077; 2025‐0955; 2025‐1159). The research was conducted in accordance with the guidelines of the Declaration of Helsinki. All participants voluntarily enrolled in the study and signed informed consent forms; moreover, they retained the right to withdraw at any time.

## 4 Results

### 4.1 Participants' Characteristics

A total of 147 HD patients were screened for eligibility. Of these, 46 did not meet the inclusion criteria, and 45 declined to participate. Consequently, 56 participants consented to participate and were allocated to the multicomponent exercise intervention group (*n* = 28) or the control group (*n* = 28) according to the randomisation of their fixed haemodialysis‐schedule clusters. During the study period, four participants in the intervention group and two in the control group withdrew, resulting in 50 completers (an 89.3% retention rate). The study flow diagram is presented in Figure 1.

**FIGURE 1:**
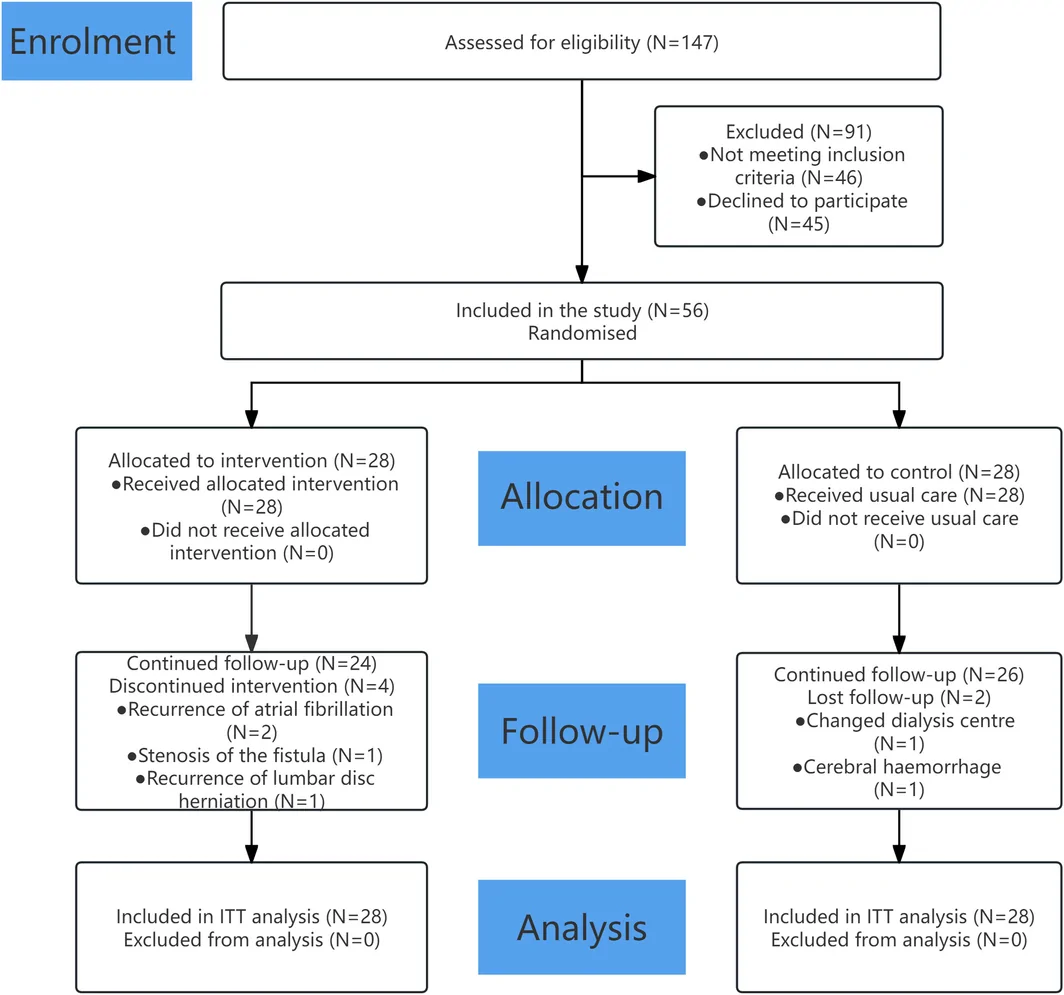
CONSORT flow diagram.

The mean age was 62.79 years (standard deviation [SD] = 7.51) in the intervention group and 69.93 years (SD = 4.59) in the control group (*p* < 0.001). No significant differences were observed in other sociodemographic characteristics, clinical features, or primary and secondary outcomes except for age. Comparisons of baseline characteristics are shown in Table 2.

**TABLE 2:** Baseline characteristics of participants.

Characteristic	Overall (*N* = 56)	Control (*N* = 28)	Intervention (*N* = 28)	*p*
Age, years, mean (SD)	66.36 (7.15)	69.93 (4.59)	62.79 (7.51)	< 0.001
Sex, *n* (%)				
Male	27 (48)	16 (57)	11 (39)	0.18
Female	29 (52)	12 (43)	17 (61)	
Marital status, *n* (%)				
Married	53 (95)	27 (96)	26 (93)	> 0.99
Unmarried	3 (5.4)	1 (3.6)	2 (7.1)	
Education, *n* (%)				
Primary	14 (25)	10 (36)	4 (14)	0.11
Secondary	20 (36)	10 (36)	10 (36)	
High school	15 (27)	7 (25)	8 (29)	
College or above	7 (13)	1 (3.6)	6 (21)	
Sarcopenia, *n* (%)				
Possible sarcopenia	18 (32)	10 (36)	8 (29)	0.57
Confirmed sarcopenia	38 (68)	18 (64)	20 (71)	
Primary kidney disease, *n* (%)				
Glomerulonephritis	39 (70)	18 (64)	21 (75)	0.60
Diabetes	9 (16)	6 (21)	3 (11)	
Others	8 (14)	4 (14)	4 (14)	
History of CVD, *n* (%)	23 (41)	14 (50)	9 (32)	0.17
History of diabetes, *n* (%)	15 (27)	7 (25)	8 (29)	0.76
Dialysis duration, months, median (IQR)	86.50 (40.00–156.00)	79.50 (21.50–153.50)	109.00 (47.50–171.50)	0.40
Handgrip strength, kg, median (IQR)	20.45 (16.65–25.80)	22.10 (15.95–25.45)	19.90 (16.65–26.85)	0.77
Gait speed, m/s, mean (SD)	0.90 (0.17)	0.86 (0.20)	0.94 (0.13)	0.16
SPPB scores, median (IQR)	9 (8–10)	9 (8–9)	9 (8–11)	0.21
SMI, kg/m^2^, mean (SD)	6.09 (1.05)	6.29 (1.02)	5.88 (1.06)	0.18
Symptoms and problems subscale, median (IQR)	77.08 (66.67–83.33)	77.08 (72.92–83.33)	77.08 (63.55–83.33)	0.72
Effects of kidney disease on daily life subscale, mean (SD)	60.21 (16.47)	60.49 (13.24)	59.94 (19.41)	0.85
Burden of kidney disease subscale, median (IQR)	37.50 (18.75–56.25)	37.50 (18.75–50.00)	43.75 (25.00–56.25)	0.56
PCS subscale, mean (SD)	40.47 (7.05)	40.12 (5.60)	40.81 (8.35)	0.67
MCS subscale, mean (SD)	41.80 (8.17)	40.90 (7.10)	42.70 (9.16)	0.32
CRP, mg/L, median (IQR)	2.35 (0.90–5.14)	2.77 (1.13–5.63)	2.23 (0.69–4.38)	0.37

Abbreviations: CRP, C‐reactive protein; CVD, cardiovascular disease; IQR, interquartile range; MCS, mental component summary; PCS, physical component summary; SD, standard deviation; SMI, skeletal muscle mass index; SPPB, short physical performance battery.

### 4.2 Primary Outcomes

After adjustment for age, sex and dialysis duration, handgrip strength was higher in the intervention group than in the control group at Week 4 (mean difference [MD]: 2.95 kg, 95% confidence interval [CI]: 0.753–5.144, raw *p* = 0.009; Cohen's *d* = 0.487), Week 8 (MD: 2.83 kg, 95% CI: 0.614–5.046, raw *p* = 0.013; Cohen's *d* = 0.467) and Week 12 (MD: 3.03 kg, 95% CI: 0.820–5.244, raw *p* = 0.008; Cohen's *d* = 0.500). However, these between‐group differences were no longer statistically significant after Bonferroni correction and did not reach the minimal clinically important difference of 5 kg (Bohannon 2019). Similarly, the group × time interaction contrasts for handgrip strength were nominally significant at Weeks 4, 8 and 12 before correction, but none remained statistically significant after Bonferroni correction.

At Weeks 4 and 8, no statistically significant between‐group differences in gait speed were observed. At Week 12, the intervention group had a greater gait speed than the control group (MD: 0.152 m/s; 95% CI: 0.034–0.271; raw *p* = 0.013; Cohen's *d* = 0.831), and the magnitude of this difference exceeded the minimal clinically important difference of 0.1 m/s (Perera et al. 2006). However, this between‐group difference was no longer statistically significant after Bonferroni correction. The group × time interaction contrast for gait speed at Week 12 was also nominally significant before correction, but this effect was not retained after Bonferroni correction.

For SPPB scores, no statistically significant between‐group differences were observed at Weeks 4 and 8. At Week 12, SPPB scores were higher in the intervention group than in the control group (MD: 1.289; 95% CI: 0.145–2.433; raw *p* = 0.028; Cohen's *d* = 0.704), and the magnitude of this difference exceeded the minimal clinically important difference of 1 point (Perera et al. 2006). However, this between‐group difference was no longer statistically significant after Bonferroni correction. The group × time interaction contrasts for SPPB scores were not statistically significant at any time point, either before or after Bonferroni correction.

No statistically significant between‐group difference in SMI was observed at Week 12 (raw *p =* 0.593). Although the group × time interaction contrast for SMI was nominally significant before correction (raw *p =* 0.019), this effect was no longer statistically significant after Bonferroni correction. The results are shown in Figure 2 and Table 3. The model selection, fit indices and diagnostics for primary outcomes are presented in Table S1.

**FIGURE 2:**
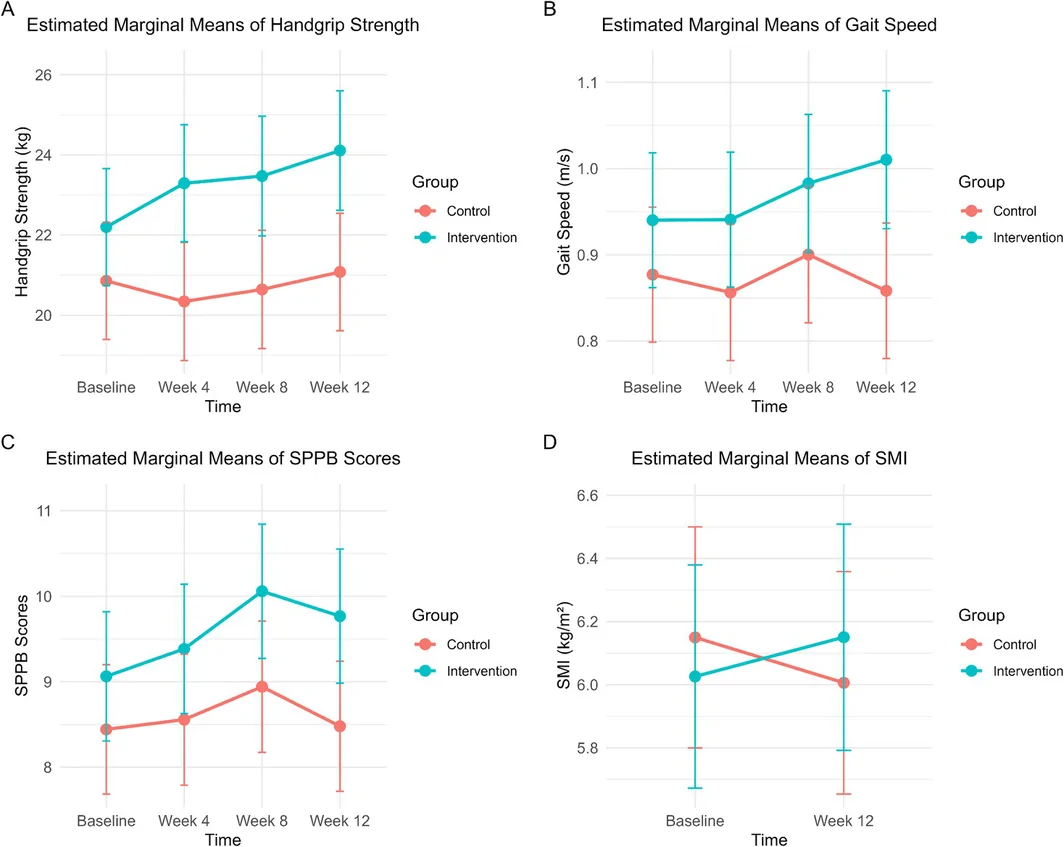
Estimated marginal means of primary outcomes across specified time points for the intervention and control groups. SMI, skeletal muscle mass index; SPPB, short physical performance battery. Data are presented as estimated marginal means from linear mixed models.

**TABLE 3:** Between‐group comparison of primary outcomes across specified time points.

Outcome	Time	Intervention (*n* = 28)	Control (*n* = 28)	Intervention effect				Time×group interaction		
		Mean (SE)	Mean (SE)	MD (95% CI)	Raw *p*	Adjusted *p*	Cohen's *d*	*β* (95% CI)	Raw *p*	Adjusted *p*
Handgrip strength (kg)	Baseline	22.20 (0.73)	20.86 (0.73)	1.342 (−0.849, 3.533)						
	Week 4	23.29 (0.73)	20.34 (0.74)	2.948 (0.753, 5.144)	0.009	0.091	0.487	1.607 (0.305, 2.908)	0.016	0.155
	Week 8	23.47 (0.75)	20.64 (0.74)	2.830 (0.614, 5.046)	0.013	0.129	0.467	1.489 (0.150, 2.827)	0.029	0.293
	Week 12	24.11 (0.75)	21.08 (0.74)	3.032 (0.820, 5.244)	0.008	0.078	0.500	1.690 (0.361, 3.020)	0.013	0.127
Gait speed (m/s)	Baseline	0.94 (0.04)	0.88 (0.04)	0.063 (−0.054, 0.181)						
	Week 4	0.94 (0.04)	0.86 (0.04)	0.085 (−0.033, 0.202)	0.157	1.000	0.462	0.021 (−0.050, 0.092)	0.556	1.000
	Week 8	0.98 (0.04)	0.90 (0.04)	0.083 (−0.036, 0.201)	0.170	1.000	0.451	0.019 (−0.054, 0.092)	0.603	1.000
	Week 12	1.01 (0.04)	0.86 (0.04)	0.152 (0.034, 0.271)	0.013	0.125	0.831	0.089 (0.016, 0.161)	0.016	0.163
SPPB scores	Baseline	9.06 (0.38)	8.44 (0.38)	0.622 (−0.503, 1.746)						
	Week 4	9.39 (0.38)	8.56 (0.39)	0.826 (−0.303, 1.955)	0.150	1.000	0.451	0.204 (−0.714, 1.123)	0.663	1.000
	Week 8	10.06 (0.40)	8.94 (0.39)	1.117 (−0.031, 2.265)	0.056	0.564	0.610	0.496 (−0.448, 1.439)	0.303	1.000
	Week 12	9.77 (0.40)	8.48 (0.38)	1.289 (0.145, 2.433)	0.028	0.276	0.704	0.667 (−0.270, 1.605)	0.163	1.000
SMI (kg/m^2^)	Baseline	6.03 (0.18)	6.15 (0.17)	−0.124 (−0.656, 0.408)						
	Week 12	6.15 (0.18)	6.01 (0.18)	0.144 (−0.392, 0.681)	0.593	1.000	0.142	0.268 (0.044, 0.492)	0.019	0.188

*Note:* Adjusted for age, sex and dialysis duration. Values are estimated marginal means from linear mixed models. Bonferroni‐adjusted *p* values were calculated for the ten primary comparisons. The 95% confidence intervals shown were not adjusted for multiple comparisons. For primary outcomes, statistical significance was determined using Bonferroni‐adjusted *p* values, with adjusted *p* < 0.05 considered statistically significant.

Abbreviations: CI, confidence interval; MD, mean difference; SE, standard error; SMI, skeletal muscle mass index; SPPB, short physical performance battery.

### 4.3 Secondary Outcomes

Compared with the control group, the intervention group showed no significant improvements in KDQOL‐36 scores or CRP levels. Although some KDQOL‐36 scores showed numerically favourable changes in the intervention group, all confidence intervals crossed zero, and no statistically significant between‐group differences were observed. No statistically significant group × time interactions were detected for KDQOL‐36 scores or CRP levels. The results are shown in Table 4. The model selection, fit indices and diagnostics for secondary outcomes are presented in Table S2.

**TABLE 4:** Between‐group comparison of secondary outcomes across specified time points.

Outcome	Time	Intervention (*n* = 28)	Control (*n* = 28)	Intervention effect			Time×group interaction	
		Mean (SE)	Mean (SE)	MD (95% CI)	Raw *p*	Cohen's *d*	*β* (95% CI)	Raw *p*
Symptoms and problems subscale	Baseline	74.031 (2.164)	77.564 (2.165)	−3.533 (−9.883, 2.816)				
	Week 4	80.130 (2.164)	82.123 (2.207)	−1.993 (−8.377, 4.392)	0.538	−0.162	1.541 (−4.370, 7.451)	0.610
	Week 8	81.615 (2.273)	84.205 (2.207)	−2.590 (−9.110, 3.930)	0.433	−0.211	0.943 (−5.124, 7.010)	0.761
	Week 12	85.220 (2.273)	84.799 (2.183)	0.422 (−6.072, 6.915)	0.898	0.034	3.955 (−2.075, 9.984)	0.200
Effects of kidney disease on daily life subscale	Baseline	59.271 (3.580)	61.111 (3.581)	−1.840 (−12.412, 8.733)				
	Week 4	62.062 (3.580)	55.571 (3.639)	6.491 (−4.127, 17.109)	0.228	0.394	8.330 (−0.429, 17.090)	0.064
	Week 8	66.286 (3.729)	59.349 (3.639)	6.937 (−3.865, 17.738)	0.206	0.421	8.776 (−0.222, 17.774)	0.058
	Week 12	62.907 (3.729)	62.640 (3.606)	0.267 (−10.498, 11.033)	0.961	0.016	2.107 (−6.835, 11.048)	0.645
Burden of kidney disease subscale	Baseline	40.009 (4.124)	39.783 (4.124)	0.226 (−11.891, 12.343)				
	Week 4	42.018 (4.124)	42.242 (4.202)	−0.224 (−12.404, 11.956)	0.971	−0.010	−0.450 (−11.444, 10.545)	0.936
	Week 8	47.980 (4.322)	41.521 (4.202)	6.459 (−5.968, 18.887)	0.306	0.292	6.233 (−5.054, 17.520)	0.281
	Week 12	44.447 (4.322)	44.386 (4.157)	0.060 (−12.318, 12.439)	0.992	0.003	−0.165 (−11.383, 11.052)	0.977
MCS subscale	Baseline	44.421 (1.449)	41.439 (1.450)	2.982 (−1.322, 7.285)				
	Week 4	44.687 (1.449)	40.989 (1.469)	3.697 (−0.620, 8.015)	0.092	0.528	0.716 (−2.445, 3.876)	0.658
	Week 8	45.671 (1.498)	41.480 (1.469)	4.191 (−0.186, 8.568)	0.060	0.599	1.209 (−2.039, 4.458)	0.467
	Week 12	46.502 (1.498)	42.551 (1.458)	3.951 (−0.414, 8.317)	0.076	0.565	0.969 (−2.258, 4.197)	0.557
PCS subscale	Baseline	42.157 (1.304)	40.081 (1.304)	2.076 (−1.791, 5.944)				
	Week 4	42.587 (1.304)	40.463 (1.323)	2.124 (−1.757, 6.005)	0.280	0.334	0.048 (−2.877, 2.973)	0.974
	Week 8	43.959 (1.350)	40.511 (1.323)	3.447 (−0.490, 7.385)	0.086	0.542	1.371 (−1.635, 4.377)	0.373
	Week 12	44.128 (1.350)	40.839 (1.312)	3.290 (−0.637, 7.216)	0.100	0.517	1.213 (−1.774, 4.200)	0.427
CRP (mg/L)	Baseline	3.002 (0.776)	4.215 (0.759)	−1.213 (−3.489, 1.063)				
	Week 12	3.021 (0.829)	3.418 (0.770)	−0.397 (−2.745, 1.951)	0.738	−0.111	0.816 (−1.533, 3.165)	0.499

*Note:* Adjusted for age, sex and dialysis duration. Values are estimated marginal means from linear mixed models. Secondary outcomes were exploratory and were analysed without multiplicity correction; raw *p* values and 95% confidence intervals were interpreted descriptively.

Abbreviations: CI, confidence interval; CRP, C‐reactive protein; MCS, mental component summary; MD, mean difference; PCS, physical component summary; SE, standard error.

### 4.4 Exercise Adherence

In the intervention group, 24 out of 28 participants (85.7%) completed the 12‐week intervention. The mean overall adherence was 83.7% ± 22.0%, compared with 91.1% ± 12.5% among those who completed the study, as shown in Table S3.

### 4.5 Adverse Events

Four participants in the intervention group withdrew during follow‐up. These withdrawals were due to one case of fistula stenosis, one recurrence of lumbar disc herniation and two recurrences of atrial fibrillation. After medical evaluation, these events were considered unrelated to the intervention. No serious intervention‐related adverse events occurred. The most common exercise‐related symptom was mild muscle soreness, which resolved with rest and required no specific treatment.

### 4.6 Sensitivity Analysis

The PP sensitivity analyses were consistent with the ITT findings for all outcomes, with absolute differences < 0.2 × baseline SD. Exploratory cluster‐level ICC estimates were small (range: 0.000–0.079), indicating low within‐cluster correlation; however, owing to the large average cluster size (*n* ≈ 28), the estimated design effect remained non‐negligible (range: 1.00–3.14). Detailed time‐point assessments and Week‐12 summary results are provided in Tables S4 and S5.

## 5 Discussion

This study showed that a nurse‐led, multicomponent exercise intervention was associated with favourable trends in handgrip strength, gait speed and SPPB scores in HD patients with possible or confirmed sarcopenia; however, these between‐group differences were not statistically significant after Bonferroni correction. Furthermore, the increase in grip strength did not reach the MCID of 5 kg. No statistically significant differences were observed in the SMI, KDQOL‐36 scores or CRP level. Although the improvements in the intervention group were modest, the effect sizes for handgrip strength, gait speed and SPPB scores were moderate to large, and the direction of effect consistently favoured the intervention, suggesting potential clinical relevance. The good adherence and absence of serious intervention‐related adverse events further support the feasibility and apparent safety of the nurse‐led, multicomponent exercise intervention.

Nurses are present at the bedside during HD sessions and can provide continuous supervision, real‐time feedback, prompt handling of adverse events, and personalised support and encouragement (Junqué‐Jiménez et al. 2023). Compared with interventions led by exercise specialists or patients themselves, nurse‐led interventions are more accessible, sustainable and patient‐centred. This may partly explain the high adherence and favourable safety profile observed in this study. This nurse‐led intervention is low‐cost and does not require additional specialised personnel or exercise facilities, making it particularly suitable for clinical settings where access to exercise specialists is limited. These characteristics may support its future implementation in HD clinical practice, pending confirmation in larger studies.

Multicomponent exercise may be a reasonable exercise strategy for HD patients with sarcopenia because it is intended to address several interrelated aspects of physical function including muscle strength, aerobic capacity, flexibility, balance and postural stability (Izquierdo et al. 2025; Sayer et al. 2024; Wilkinson et al. 2025). These domains are particularly relevant in HD patients, who often experience reduced physical function and increased fall risk. However, the present study compared nurse‐led multicomponent exercise with standard care only and did not include single‐modality exercise, resistance‐only exercise, aerobic‐only exercise, or combined resistance and aerobic exercise control groups. Therefore, the findings should not be interpreted as evidence that multicomponent exercise is superior to other exercise prescriptions. Future adequately powered multi‐arm trials are needed to directly compare multicomponent exercise with single‐modality and combined resistance‐aerobic exercise programmes in HD patients with sarcopenia.

Unlike previous studies (Monti et al. 2023; Sáez de Asteasu et al. 2024), our study found that although handgrip strength, gait speed and SPPB scores showed numerically favourable changes in the intervention group, these between‐group differences were no longer statistically significant after Bonferroni correction for multiple comparisons. This may be partly attributable to the limited sample size of our study, which was calculated on the basis of handgrip strength alone without adjustment for multiple primary outcomes, and the conservative Bonferroni correction method adopted. Although this approach controls the family‐wise error rate (FWER), it also reduces statistical power (Vickerstaff et al. 2019). Therefore, we cannot rule out the possibility that a larger sample size might have yielded statistically significant improvements in handgrip strength, gait speed and SPPB scores.

Compared with the control group, the intervention group did not show a significant improvement in SMI after 12 weeks, which is consistent with previous findings (Chaovarin et al. 2024). The absence of an increase in SMI despite improved muscle strength may be explained by several mechanisms. First, in the early stages of exercise intervention, muscle strength improvement is driven mainly by neural adaptations, whereas skeletal muscle hypertrophy usually requires 3–6 months of continuous exercise (Hughes et al. 2018). Second, the low‐to‐moderate intensity resistance exercise used in this study (Borg 11–13) can improve neuromuscular function, but the intensity may be insufficient to significantly promote muscle hypertrophy (Izquierdo et al. 2025). Furthermore, the chronic inflammatory state of HD patients may inhibit muscle anabolic responses (Wang et al. 2022), which is consistent with the lack of significant changes in CRP levels observed in this study.

Our study did not show significant improvements in the KDQOL‐36 scores, which is consistent with findings from other studies (Greenwood et al. 2021; Nakoui et al. 2025). Exercise stimulates the production of neurotransmitters and monoamines in the hippocampus by modulating the expression of brain‐derived neurotrophic factors, thereby exerting antidepressant and anxiolytic effects, improving mood and reducing fatigue. However, these beneficial effects of exercise may require more than 12 weeks to manifest (Soong et al. 2025). The KDQOL‐36 instrument assesses multiple dimensions, including physical and mental health, symptom burden, and the impact of kidney disease on daily activities. Compared with physical functioning, patients may be more likely to be affected by the burden of dialysis treatment, dietary restrictions, work, social interactions and financial difficulties (Traise et al. 2025). Furthermore, patient‐reported outcomes, such as those itemised in the KDQOL‐36, are subjective and dynamic, as participants' responses to each question can vary at different time points (Lapin 2020). Consequently, a 12‐week exercise intervention programme may be insufficient to improve these outcomes.

Consistent with the findings of previous studies (Nakoui et al. 2025), our study did not show significant improvements in C‐reactive protein levels. A previous systematic review suggested that reductions in CRP may be more apparent after long‐term resistance exercise performed at higher Borg RPE levels (> 15) (Meléndez Oliva et al. 2022). Therefore, the 12‐week duration of the multicomponent exercise intervention in this study and the low‐to‐moderate exercise intensity (Borg 11–13) may not have been sufficient to influence CRP levels. Studies with longer durations and higher exercise intensities are warranted to better understand the effects of multicomponent exercise on inflammatory mediators in HD patients.

This study has several limitations that warrant consideration. First, the 12‐week follow‐up period was too short to evaluate the long‐term effects of the multicomponent exercise intervention. Second, the sample size was calculated on the basis of handgrip strength alone, without adjustment for multiple primary outcomes or the design effect associated with cluster allocation, which may have reduced statistical power after Bonferroni correction. Third, cluster randomisation was performed on the basis of fixed HD schedules without individual‐level randomisation or stratification, which may have introduced unmeasured confounding associated with HD schedules. In addition, no subgroup analyses by exercise type were performed, and the absence of separate aerobic and resistance control groups meant that the relative contribution of each component could not be determined. Despite these limitations, the findings may inform the development of feasible nurse‐led exercise interventions for HD patients with sarcopenia.

## 6 Conclusion

In summary, the 12‐week nurse‐led, multicomponent exercise intervention did not produce statistically significant between‐group improvements in the primary outcomes after Bonferroni correction, nor did it significantly improve the secondary outcomes in HD patients with possible or confirmed sarcopenia. Nevertheless, changes in handgrip strength, gait speed and SPPB scores consistently favoured the intervention group and showed moderate‐to‐large effect sizes. The intervention was also associated with good adherence, and no serious intervention‐related adverse events were reported. These findings suggest that a nurse‐led, multicomponent exercise programme is feasible and appears safe in this population, and may have favourable potential effects on muscle strength and physical performance. Larger, adequately powered, multi‐arm randomised trials with stratified individual‐level randomisation, longer follow‐up periods and appropriate exercise comparator groups are warranted to further evaluate the potential effects of nurse‐led multicomponent exercise and clarify the relative contribution of different exercise components.

## Author Contributions

Genlian Cai: original draft, visualisation, investigation, supervision, project administration, funding acquisition, conceptualization. Liuqian Zhang, Jing Zhang and Jinjin Zhou: supervision, project administration, methodology, conceptualization. Yujiao Zhang and Bingxin Zhan: data collection, curation and analysis. Qi Guo: review and editing, supervision. Jinping Ying: review and editing, supervision, project administration and methodology.

## Funding

This work was supported by the Medical and Health Research Project of Zhejiang Province (2023KY101) and Discipline Construction Project Fund for Nursing Research (FAHZU no. 2022ZYHL003).

## Ethics Statement

This study was approved by the Institutional Review Board of the First Affiliated Hospital of Zhejiang University School of Medicine (approval nos. 2022‐437; 2024‐077; 2025‐0955 and 2025‐1159).

## Consent

All of the participants voluntarily enrolled in the study and signed informed consent forms; moreover, they retained the right to withdraw at any time.

## Conflicts of Interest

The authors declare no conflicts of interest.

## Supporting information


**Table S1:** Linear mixed model selection, fit indices and residual diagnostics for primary outcomes.

**Table S2:** Linear mixed model selection, fit indices and residual diagnostics for secondary outcomes.

**Table S3:** Individual exercise adherence by participant (*n* = 28).

**Table S4:** ITT and PP analyses by assessment time point.

**Table S5:** Sensitivity analyses for treatment effects at Week 12 for primary and secondary outcomes.

## Data Availability

The data that support the findings of this study are available from the corresponding author upon reasonable request.
