# Supplementary material for: Effects of a Nurse‐Led Multicomponent Exercise Intervention for Haemodialysis Patients With Possible or Confirmed Sarcopenia: A Cluster Randomised Trial
Source: Nurs Open. 2026 Oct 30;13(11):e70651. doi: 10.1002/nop2.70651 (PMC13722802; doi:10.1002/nop2.70651)
Supplement: Supplementary file 1 [file NOP2-13-e70651-s001.docx]

| **Table S4. ITT and PP analyses by assessment time point.** | | | | | | | |
| --- | --- | --- | --- | --- | --- | --- | --- |
| **Outcome** | **Timepoint** | **ITT N** | **ITT Estimate (95% CI)** | **P value** | **PP N** | **PP Estimate (95% CI)** | **P value** |
| SPPB **Scores** | Week 4 | 56 | +0.826 (-0.303, +1.955) | 0.150 | 50 | +0.922 (-0.225, +2.069) | 0.118 |
|  | Week 8 | 56 | +1.117 (-0.031, +2.265) | 0.056 | 50 | +1.162 (+0.016, +2.309) | 0.050 |
|  | Week 12 | 56 | +1.289 (+0.145, +2.433) | 0.028 | 50 | +1.217 (+0.070, +2.364) | 0.040 |
| Gait Speed | Week 4 | 56 | +0.085 (-0.033, +0.202) | 0.157 | 50 | +0.093 (-0.026, +0.212) | 0.129 |
|  | Week 8 | 56 | +0.083 (-0.036, +0.201) | 0.170 | 50 | +0.088 (-0.031, +0.206) | 0.152 |
|  | Week 12 | 56 | +0.152 (+0.034, +0.271) | 0.013 | 50 | +0.157 (+0.038, +0.276) | 0.011 |
| Handgrip strength | Week 4 | 56 | +2.948 (+0.753, +5.144) | 0.009 | 50 | +3.163 (+0.925, +5.402) | 0.007 |
|  | Week 8 | 56 | +2.830 (+0.614, +5.046) | 0.013 | 50 | +2.926 (+0.687, +5.164) | 0.012 |
|  | Week 12 | 56 | +3.032 (+0.820, +5.244) | 0.008 | 50 | +3.071 (+0.832, +5.309) | 0.009 |
| PCS subscale | Week 4 | 56 | +2.124 (-1.757, +6.005) | 0.280 | 50 | +1.868 (-2.096, +5.832) | 0.358 |
|  | Week 8 | 56 | +3.447 (-0.490, +7.385) | 0.086 | 50 | +3.157 (-0.807, +7.121) | 0.122 |
|  | Week 12 | 56 | +3.290 (-0.637, +7.216) | 0.100 | 50 | +2.851 (-1.113, +6.815) | 0.162 |
| MCS subscale | Week 4 | 56 | +3.697 (-0.620, +8.015) | 0.092 | 50 | +3.776 (-0.626, +8.177) | 0.096 |
|  | Week 8 | 56 | +4.191 (-0.186, +8.568) | 0.060 | 50 | +4.043 (-0.358, +8.445) | 0.075 |
|  | Week 12 | 56 | +3.951 (-0.414, +8.317) | 0.076 | 50 | +3.677 (-0.725, +8.078) | 0.105 |
| Symptoms and Problems subscale | Week 4 | 56 | -1.993 (-8.377, +4.392) | 0.538 | 50 | -0.954 (-7.511, +5.603) | 0.776 |
|  | Week 8 | 56 | -2.590 (-9.110, +3.930) | 0.433 | 50 | -2.340 (-8.897, +4.217) | 0.486 |
|  | Week 12 | 56 | +0.422 (-6.072, +6.915) | 0.898 | 50 | +0.785 (-5.773, +7.342) | 0.815 |
| Effects of Kidney Disease on Daily Life subscale | Week 4 | 56 | +6.491 (-4.127, +17.109) | 0.228 | 50 | +7.768 (-3.203, +18.739) | 0.168 |
|  | Week 8 | 56 | +6.937 (-3.865, +17.738) | 0.206 | 50 | +7.766 (-3.205, +18.738) | 0.168 |
|  | Week 12 | 56 | +0.267 (-10.498, +11.033) | 0.961 | 50 | +1.032 (-9.939, +12.003) | 0.854 |
| Burden of Kidney Disease subscale | Week 4 | 56 | -0.224 (-12.404, +11.956) | 0.971 | 50 | +1.775 (-10.752, +14.303) | 0.782 |
|  | Week 8 | 56 | +6.459 (-5.968, +18.887) | 0.306 | 50 | +7.965 (-4.562, +20.493) | 0.215 |
|  | Week 12 | 56 | +0.060 (-12.318, +12.439) | 0.992 | 50 | +1.307 (-11.221, +13.835) | 0.838 |
| SMI | Week 12 | 56 | +0.144 (-0.392, 0.681) | 0.593 | 50 | +0.153 (-0.393, 0.699) | 0.584 |
| CRP | Week 12 | 56 | -0.397 (-2.745, 1.951) | 0.738 | 50 | -0.422 (-2.824, 1.980) | 0.731 |
| Note: Estimates are adjusted for age, sex, dialysis duration. Method: estimated marginal means from LMM (emmeans); SMI: Skeletal Muscle Mass Index; PCS: Physical Component Summary; MCS: Mental Component Summary; SPPB: Short Physical Performance Battery; CRP: C-reactive protein. | | | | | | | |
