# Supplementary material for: Effects of a Nurse‐Led Multicomponent Exercise Intervention for Haemodialysis Patients With Possible or Confirmed Sarcopenia: A Cluster Randomised Trial
Source: Nurs Open. 2026 Oct 30;13(11):e70651. doi: 10.1002/nop2.70651 (PMC13722802; doi:10.1002/nop2.70651)
Supplement: Supplementary file 2 [file NOP2-13-e70651-s002.docx]

| **Table S5. Sensitivity analyses for treatment effects at week 12 for primary and secondary outcomes.** | | | | | | | | | | | | |
| --- | --- | --- | --- | --- | --- | --- | --- | --- | --- | --- | --- | --- |
| **Outcome** | **Total N** | **Completers n (%)** | **Raw Effect (DID)** | **ITT Estimate (95% CI)** | **ITT P value** | **PP Estimate (95% CI)** | **PP P value** | **ICC** | **Design Effect** | **Baseline P** | **Covariates** | **ITT vs. PP** |
| SPPB **Scores** | 56 | 50 (89.3%) | +0.516 | +1.289 (+0.145, +2.433) | 0.028 | +1.217 (+0.070, +2.364) | 0.040 | 0.056 | 2.51 | 0.309 | Age, sex, dialysis duration | Consistent |
| Gait Speed | 56 | 50 (89.3%) | +0.097 | +0.152 (+0.034, +0.271) | 0.013 | +0.157 (+0.038, +0.276) | 0.011 | 0.079 | 3.14 | 0.302 | Age, sex, dialysis duration | Consistent |
| Handgrip strength | 56 | 50 (89.3%) | +1.650 | +3.032 (+0.820, +5.244) | 0.008 | +3.071 (+0.832, +5.309) | 0.009 | 0.000 | 1.00 | 0.138 | Age, sex, dialysis duration | Consistent |
| PCS subscale | 56 | 50 (89.3%) | +1.160 | +3.290 (-0.637, +7.216) | 0.100 | +2.851 (-1.113, +6.815) | 0.162 | 0.011 | 1.30 | 0.308 | Age, sex, dialysis duration | Consistent |
| MCS subscale | 56 | 50 (89.3%) | +1.104 | +3.951 (-0.414, +8.317) | 0.076 | +3.677 (-0.725, +8.078) | 0.105 | 0.033 | 1.89 | 0.189 | Age, sex, dialysis duration | Consistent |
| Symptoms and Problems subscale | 56 | 50 (89.3%) | +4.107 | +0.422 (-6.072, +6.915) | 0.898 | +0.785 (-5.773, +7.342) | 0.815 | 0.007 | 1.18 | 0.523 | Age, sex, dialysis duration | Consistent |
| Effects of Kidney Disease on Daily Life subscale | 56 | 50 (89.3%) | +1.282 | +0.267 (-10.498, +11.033) | 0.961 | +1.032 (-9.939, +12.003) | 0.854 | 0.000 | 1.00 | 0.869 | Age, sex, dialysis duration | Consistent |
| Burden of Kidney Disease subscale | 56 | 50 (89.3%) | -1.550 | +0.060 (-12.318, +12.439) | 0.992 | +1.307 (-11.221, +13.835) | 0.838 | 0.000 | 1.00 | 0.852 | Age, sex, dialysis duration | Consistent |
| SMI | 56 | 50 (89.3%) | +0.264 | +0.144 (-0.392, 0.681) | 0.593 | +0.153 (-0.393, +0.699) | 0.584 | 0.032 | 1.87 | 0.787 | Age, sex, dialysis duration | Consistent |
| CRP | 56 | 50 (89.3%) | +0.888 | -0.397 (-2.745, 1.951) | 0.738 | -0.422 (-2.824, +1.980) | 0.731 | 0.001 | 1.02 | 0.342 | Age, sex, dialysis duration | Consistent |
| Note: Raw Effect = unadjusted difference-in-differences (Intervention change minus Control change) among completers; ITT = intention-to-treat LMM with random intercept; PP = per-protocol completers only; Consistency defined as absolute difference < 0.2 × baseline SD. Positive values favor intervention; SMI and CRP measured at baseline and Week 12 only. SMI, Skeletal Muscle Mass Index; PCS, Physical Component Summary; MCS, Mental Component Summary; SPPB, Short Physical Performance Battery; CRP, C-reactive protein. The ICC and design effect in this table refer to exploratory cluster-level estimates for assessing the potential impact of cluster randomisation, and differ from the participant-level ICCs derived from repeated-measures LMMs in Tables S1 and S2. Baseline p indicates the p value for between-group comparison at baseline. | | | | | | | | | | | | |
