# Supplementary material for: Effects of a Nurse‐Led Multicomponent Exercise Intervention for Haemodialysis Patients With Possible or Confirmed Sarcopenia: A Cluster Randomised Trial
Source: Nurs Open. 2026 Oct 30;13(11):e70651. doi: 10.1002/nop2.70651 (PMC13722802; doi:10.1002/nop2.70651)
Supplement: Supplementary file 3 [file NOP2-13-e70651-s003.docx]

| Table **S1. Linear mixed model selection, fit indices, and residual diagnostics for primary outcomes.** | | | | |
| --- | --- | --- | --- | --- |
| Parameter | Handgrip strength | SPPB | Gait speed | SMI |
| N (subjects) | 56 | 56 | 56 | 56 |
| N (observations) | 211 | 211 | 211 | 106 |
| Candidate models | RI vs RI+RS | RI vs RI+RS | RI vs RI+RS | RI vs RI+RS |
| Selection method | AIC (REML) | AIC (REML) | AIC (REML) | AIC (REML) |
| Selected structure | Random intercept only | Random intercept only | Random intercept only | Random intercept only |
| Selection reason | RS model failed to converge | RS model failed to converge | RS model failed to converge | RS model failed to converge |
| AIC (REML) for RI model | 1003.7 | 825.2 | -162.1 | 231.8 |
| AIC (ML) for final model | 995.7 | 805.5 | -234.4 | 202.4 |
| BIC (ML final) | 1039.2 | 849.1 | -190.8 | 226.4 |
| Marginal R² | 0.693 | 0.119 | 0.144 | 0.333 |
| Conditional R² | 0.923 | 0.605 | 0.779 | 0.918 |
| ICC (95% CI) | 0.751 (0.65–0.85) | 0.552 (0.42–0.68) | 0.742 (0.64–0.84) | 0.877 (0.82–0.93) |
| Residual SD | 1.730 | 1.222 | 0.094 | 0.286 |
| Shapiro-Wilk test p value | 0.003 | 0.208 | 0.026 | 0.940 |
| Levene's test p value | 0.803 | 0.340 | 0.188 | 0.411 |
| Note: RI, Random intercept (1\|ID); RS, Random slope (1+time\|ID); ML, Maximum likelihood; REML, Restricted maximum likelihood; ICC = Intraclass correlation coefficient; SPPB, Short Physical Performance Battery; SMI, Skeletal Muscle Mass Index. Shapiro-Wilk and Levene's tests were conducted on conditional residuals from the final random-intercept LMMs; Levene's test compared residual variances across group x timepoint cells. | | | | |
