# Supplementary material for: Effects of a Nurse‐Led Multicomponent Exercise Intervention for Haemodialysis Patients With Possible or Confirmed Sarcopenia: A Cluster Randomised Trial
Source: Nurs Open. 2026 Oct 30;13(11):e70651. doi: 10.1002/nop2.70651 (PMC13722802; doi:10.1002/nop2.70651)
Supplement: Supplementary file 4 [file NOP2-13-e70651-s004.docx]

| **Table S2. Linear mixed model selection, fit indices, and residual diagnostics for secondary outcomes.** | | | | | | |
| --- | --- | --- | --- | --- | --- | --- |
| Parameter | Symptoms and Problems  subscale | Effects of Kidney Disease on  Daily Life subscale | Burden of Kidney Disease  subscale | PCS subscale | MCS subscale | CRP |
| N (subjects) | 56 | 56 | 56 | 56 | 56 | 56 |
| N (observations) | 211 | 211 | 211 | 211 | 211 | 106 |
| Candidate models | RI vs RI+RS | RI vs RI+RS | RI vs RI+RS | RI vs RI+RS | RI vs RI+RS | RI vs RI+RS |
| Selection method | AIC (REML) | AIC (REML) | AIC (REML) | AIC (REML) | AIC (REML) | AIC (REML) |
| Selected structure | Random intercept only | Random intercept only | Random intercept only | Random intercept only | Random intercept only | Random intercept only |
| Selection reason | RS model failed to converge | RS model failed to converge | RS model failed to converge | RS model failed to converge | RS model failed to converge | RS model failed to converge |
| AIC (REML) for RI model | 1566.2 | 1740.1 | 1819.3 | 1310.8 | 1345.4 | 595.8 |
| AIC (ML) for final model | 1586.0 | 1769.8 | 1852.7 | 1317.5 | 1354.3 | 589.1 |
| BIC (ML final) | 1629.6 | 1813.5 | 1896.4 | 1361.2 | 1398.0 | 613.1 |
| Marginal R² | 0.128 | 0.059 | 0.086 | 0.065 | 0.081 | 0.040 |
| Conditional R² | 0.504 | 0.571 | 0.507 | 0.633 | 0.659 | 0.338 |
| ICC (95% CI) | 0.431 (0.30–0.56) | 0.544 (0.41–0.67) | 0.460 (0.33–0.59) | 0.608 (0.48–0.73) | 0.629 (0.51–0.75) | 0.310 (0.20–0.42) |
| Residual SD | 8.029 | 11.603 | 14.593 | 3.901 | 4.209 | 3.045 |
| Shapiro-Wilk test p value | <0.001 | 0.701 | 0.198 | 0.067 | 0.046 | <0.001 |
| Levene's test p value | 0.806 | 0.883 | 0.024 | 0.269 | 0.883 | 0.740 |
| Note: RI, Random intercept (1\|ID); RS, Random slope (1+time\|ID); ML, Maximum likelihood; REML, Restricted maximum likelihood; ICC, Intraclass correlation coefficient; MCS: Mental Component Summary; PCS: Physical Component Summary; CRP: C-reactive protein. Shapiro-Wilk and Levene's tests were conducted on conditional residuals from the final random-intercept LMMs; Levene's test compared residual variances across group x timepoint cells. | | | | | | |
