# Supplementary material for: Effects of a Nurse‐Led Multicomponent Exercise Intervention for Haemodialysis Patients With Possible or Confirmed Sarcopenia: A Cluster Randomised Trial
Source: Nurs Open. 2026 Oct 30;13(11):e70651. doi: 10.1002/nop2.70651 (PMC13722802; doi:10.1002/nop2.70651)
Supplement: Supplementary file 5 [file NOP2-13-e70651-s005.docx]

| **Table S3. Individual exercise adherence by participant (n=28)** | | | | | | |
| --- | --- | --- | --- | --- | --- | --- |
| **Participant ID** | **Status** | **Aerobic exercise** | **Upper-limb Resistance exercise** | **Lower-limb Resistance exercise** | **Flexibility exercise** | **Overall adherence** |
| 13 | **Dropout** | 36.1% (13/36) | 38.9% (14/36) | 38.9% (14/36) | 38.9% (14/36) | 38.2% (55/144) |
| 22 | **Dropout** | 44.4% (16/36) | 47.2% (17/36) | 47.2% (17/36) | 47.2% (17/36) | 46.5% (67/144) |
| 23 | **Dropout** | 33.3% (12/36) | 36.1% (13/36) | 36.1% (13/36) | 36.1% (13/36) | 35.4% (51/144) |
| 26 | **Dropout** | 33.3% (12/36) | 36.1% (13/36) | 36.1% (13/36) | 36.1% (13/36) | 35.4% (51/144) |
| 1 | Completer | 97.2% (35/36) | 97.2% (35/36) | 97.2% (35/36) | 97.2% (35/36) | 97.2% (140/144) |
| 2 | Completer | 72.2% (26/36) | 100.0% (36/36) | 100.0% (36/36) | 100.0% (36/36) | 93.1% (134/144) |
| 3 | Completer | 100.0% (36/36) | 100.0% (36/36) | 100.0% (36/36) | 100.0% (36/36) | 100.0% (144/144) |
| 4 | Completer | 83.3% (30/36) | 97.2% (35/36) | 97.2% (35/36) | 97.2% (35/36) | 93.8% (135/144) |
| 5 | Completer | 91.7% (33/36) | 91.7% (33/36) | 91.7% (33/36) | 91.7% (33/36) | 91.7% (132/144) |
| 6 | Completer | 100.0% (36/36) | 100.0% (36/36) | 66.7% (24/36) | 100.0% (36/36) | 91.7% (132/144) |
| 7 | Completer | 75.0% (27/36) | 75.0% (27/36) | 75.0% (27/36) | 75.0% (27/36) | 75.0% (108/144) |
| 8 | Completer | 100.0% (36/36) | 100.0% (36/36) | 100.0% (36/36) | 100.0% (36/36) | 100.0% (144/144) |
| 9 | Completer | 100.0% (36/36) | 97.2% (35/36) | 97.2% (35/36) | 97.2% (35/36) | 97.9% (141/144) |
| 10 | Completer | 91.7% (33/36) | 100.0% (36/36) | 100.0% (36/36) | 100.0% (36/36) | 97.9% (141/144) |
| 11 | Completer | 100.0% (36/36) | 94.4% (34/36) | 94.4% (34/36) | 94.4% (34/36) | 95.8% (138/144) |
| 12 | Completer | 97.2% (35/36) | 97.2% (35/36) | 97.2% (35/36) | 97.2% (35/36) | 97.2% (140/144) |
| 14 | Completer | 75.0% (27/36) | 66.7% (24/36) | 66.7% (24/36) | 66.7% (24/36) | 68.8% (99/144) |
| 15 | Completer | 100.0% (36/36) | 100.0% (36/36) | 100.0% (36/36) | 100.0% (36/36) | 100.0% (144/144) |
| 16 | Completer | 97.2% (35/36) | 100.0% (36/36) | 100.0% (36/36) | 100.0% (36/36) | 99.3% (143/144) |
| 17 | Completer | 100.0% (36/36) | 100.0% (36/36) | 100.0% (36/36) | 100.0% (36/36) | 100.0% (144/144) |
| 18 | Completer | 88.9% (32/36) | 97.2% (35/36) | 97.2% (35/36) | 97.2% (35/36) | 95.1% (137/144) |
| 19 | Completer | 66.7% (24/36) | 66.7% (24/36) | 66.7% (24/36) | 66.7% (24/36) | 66.7% (96/144) |
| 20 | Completer | 100.0% (36/36) | 100.0% (36/36) | 100.0% (36/36) | 100.0% (36/36) | 100.0% (144/144) |
| 21 | Completer | 100.0% (36/36) | 100.0% (36/36) | 100.0% (36/36) | 100.0% (36/36) | 100.0% (144/144) |
| 24 | Completer | 100.0% (36/36) | 100.0% (36/36) | 100.0% (36/36) | 100.0% (36/36) | 100.0% (144/144) |
| 25 | Completer | 88.9% (32/36) | 58.3% (21/36) | 58.3% (21/36) | 58.3% (21/36) | 66.0% (95/144) |
| 27 | Completer | 80.6% (29/36) | 58.3% (21/36) | 58.3% (21/36) | 58.3% (21/36) | 63.9% (92/144) |
| 28 | Completer | 100.0% (36/36) | 94.4% (34/36) | 94.4% (34/36) | 94.4% (34/36) | 95.8% (138/144) |
| Note: Overall adherence: 83.7% ± 22.0%; Completers:  91.1% ± 12.5%; Data for all 28 participants randomised to the intervention group are shown. Dropouts are highlighted. For all participants, component-specific adherence was calculated using the full planned 36 sessions for each exercise component as the denominator, and overall adherence was calculated using the full planned 144 sessions as the denominator. | | | | | | |
